# Pompe disease: the role of MRI

**DOI:** 10.1186/1471-2474-14-S2-P7

**Published:** 2013-05-29

**Authors:** C Pérez Fernández, L Bosanska, U Plöckinger, A Pöllinger

**Affiliations:** 1 Klinik für Radiologie and Klinik m.S. Hepatologie und Gastroenterologie, Charité Universitätsmedizin Berlin, Berlin, Germany

## Introduction

Glycogen storage disease type II (Pompe disease) is a rare, progressive muscle disorder with a wide range of phenotypic presentations. It is caused by an inherited deficiency of acid α-glucosidase (GAA), which leads to lysosomal glycogen accumulation in various tissues, most notably cardiac, skeletal, and smooth muscle. The gradual pathologic storage of GAA in muscle cells causes irreversible muscle damage, with different signs and symptoms, including respiratory insufficiency and muscle weakness. In Pompe disease, defining severity grades is essential for prognosis and for monitoring responses to enzyme replacement therapy (available since 2006). The purpose of this analysis was to describe the MR-imaging findings of patients with Pompe disease being treated in our institution between 2010 and 2012 (n=10).

## Results/discussion

MR-imaging techniques from skeletal musculature with special fat saturated sequences, together with noninvasive measurement of the urinary glucose tetrasaccharide biomarker, provide an excellent alternative to invasive, often risky, and insufficiently sensitive muscle biopsies. In particular, the T1-weighted turbo spin echo sequences were suitable for depicting muscle atrophy and fibro-fatty muscle degeneration.

## Conclusion

MRI techniques may be appropriately and effectively used to describe muscular changes in patients with Pompe disease.

